# Evaluation of crescent formation as a predictive marker in immunoglobulin A nephropathy: a systematic review and meta-analysis

**DOI:** 10.18632/oncotarget.17502

**Published:** 2017-04-28

**Authors:** Xue Shao, Bingjue Li, Luxi Cao, Ludan Liang, Jingjuan Yang, Yucheng Wang, Shi Feng, Cuili Wang, Chunhua Weng, Xiujin Shen, Hong Jiang, Jianghua Chen

**Affiliations:** ^1^ Kidney Disease Center, The First Affiliated Hospital, College of Medicine, Zhejiang University, Hangzhou, China; ^2^ Kidney Disease Immunology Laboratory, The Third Grade Laboratory, State Administration of Traditional Chinese Medicine of China, Hangzhou, China; ^3^ Key Laboratory of Multiple Organ Transplantation, Ministry of Health, Key Laboratory of Nephropathy, Hangzhou, China

**Keywords:** Immunoglobulin A (IgA) nephropathy, Oxford classification, crescent lesions, meta-analysis

## Abstract

The 2009 Oxford Classification of immunoglobulin A (IgA) nephropathy (IgAN) identifies four histological features as predictors of renal prognosis: mesangial hypercellularity (M), endocapillary hypercellularity (E), segmental glomerulosclerosis (S), and tubular atrophy/interstitial fibrosis (T). However, the clinical and prognostic significance of crescent formation still remains controversial. Therefore, we performed a meta-analysis to evaluate the association between crescents and kidney outcome in IgAN. A total of 20 studies published from January 2009 to July 2016 involving 5,285 patients were included after systematic searches of PubMed and EMBASE databases. Pooled results showed that crescent lesions were associated with kidney failure (HR, 1.93; 95% CI, 1.49-2.50; *P* < 0.001). IgAN patients with crescents had lower eGFR levels (SMD, -0.21; 95% CI, -0.40--0.03; *P =* 0.023); higher proteinuria levels (SMD, 0.87; 95% CI, 0.11-1.63; *P* = 0.024); a larger number of patients with M1 (RR, 1.22; 95% CI, 1.07-1.40; *P* = 0.003), E1 (RR, 4.83; 95% CI, 3.04-7.66;*P* < 0.001), S1 (RR, 1.76; 95% CI, 1.11-2.80; *P* = 0.016) and T1/2 (RR, 2.74; 95% CI, 2.10-3.57; *P* < 0.001) lesions; and received immunosuppressive therapy more frequently (RD, 0.17; 95% CI, 0.11-0.23; *P* < 0.001). Our results suggest that crescent formation represents an efficient prognostic factor associated with progression to kidney failure and thus could be considered into the new Oxford Classification.

## INTRODUCTION

Immunoglobulin A (IgA) nephropathy (IgAN) is the most common glomerulonephritis worldwide and recognized to progress to end-stage kidney disease (ESKD) in approximately 20 to 40% of patients within 10 to 20 years from onset [1, 2]. IgAN is characterized by IgA deposition in the glomerular mesangium with widely variable clinical presentations, including impaired estimate glomerular filtration rate (eGFR), arterial hypertension and proteinuria, and pathological features of renal biopsy specimens [1–3]. Histologic classification of IgAN is essential for evaluating the severity of lesions and guiding appropriate therapeutic strategies during clinical practice [2, 4]. In 2009, the Oxford Classification of IgAN was developed by an international working group to establish a consensus on identifying the specific pathologic features reliably predicting the risk of IgAN progression. By analyzing renal biopsy specimens from a cohort of 265 patients from 11 countries in four continents, this study proposed four pathological features—mesangial hypercellularity (M), endocapillary hypercellularity (E), segmental glomerulosclerosis (S), and tubular atrophy and interstitial fibrosis (T)—as the histopathologic predictor of renal outcome of IgAN, independently of clinical indicators [5, 6].

In 2014, the VALIGA study (validation of IgA nephropathy), including a cohort of 1,147 patients from 13 European countries with unrestricted criteria at entry, provided a validation of the independent predictive value of the Oxford Classification [7]. The presence of crescents in both studies was not identified as a prognostic variable, mainly due to their low prevalence in the enrolled cohort [5–7]. However, the predictive value of crescent lesions in IgAN is still debated. Several studies with the cohorts differed from Oxford and VALIGA have described crescent formation as a predictive factor in the prognosis of IgAN [8–18]. In 2013, a new Japanese histologic classification (JHC) of IgAN proposed cellular/fibrocellular crescents as significant histologic prognostic variables [19]. Therefore, our study aimed to conduct a systematic review and meta-analysis to investigate crescent formation as a predictive marker in the progression of IgAN.

## RESULTS

### Study selection and characteristics

**Figure 1:**
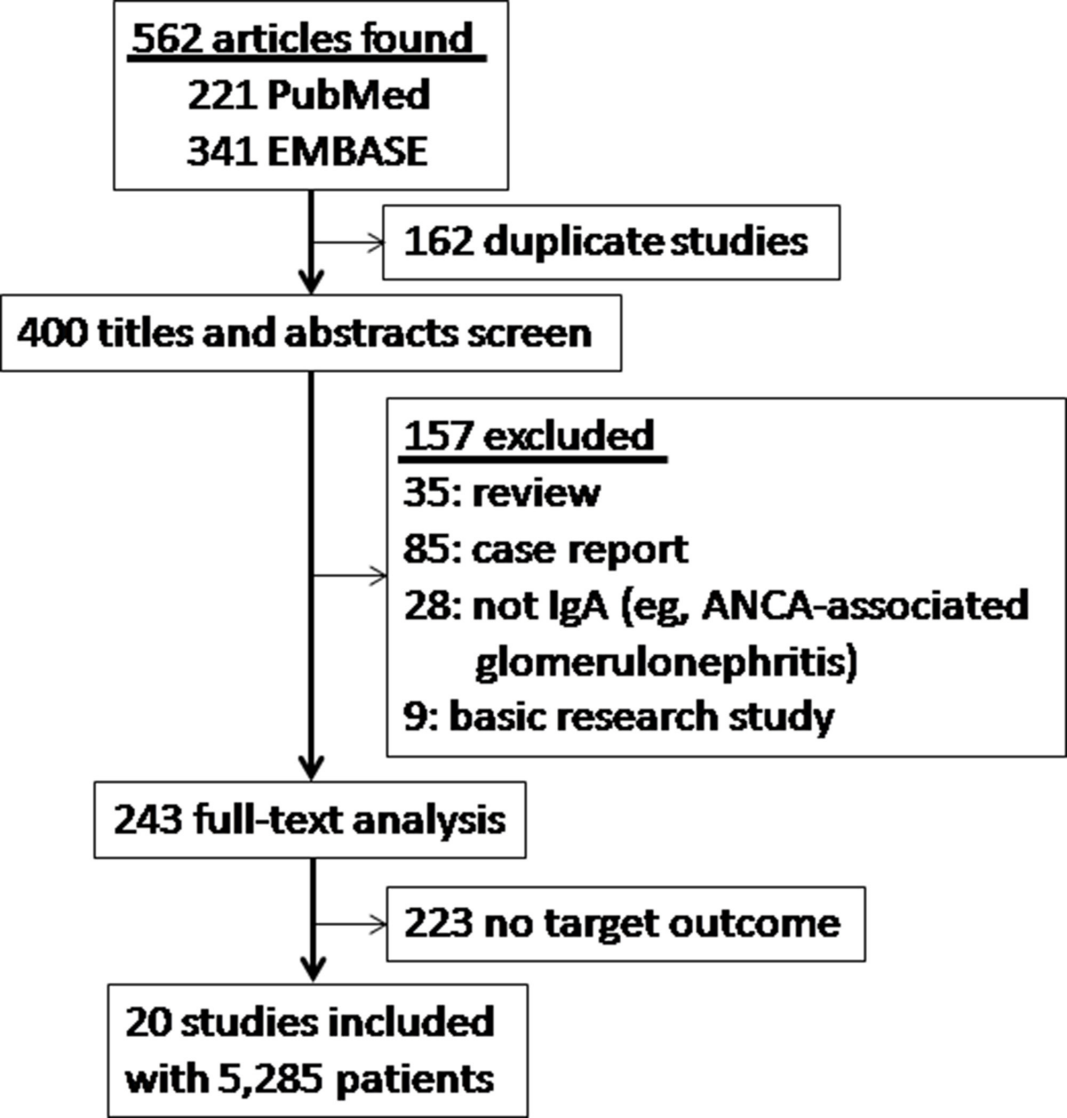
Flow chart for study selection 562 articles were downloaded as potential studies, and 162 publications of which were excluded due to duplication. After detailed evaluation, 380 more were excluded according to the inclusion and exclusion criteria. Eventually, 20 studies involving a total of 5,285 patients were included.

562 full-text articles were downloaded as potential studies, and 162 publications of which were excluded due to duplication. After detailed evaluation, 380 more were excluded according to the inclusion and exclusion criteria. Eventually, 20 studies published from 2009 to 2016 involving a total of 5,285 patients were included in this meta-analysis (Figure 1). Eleven studies [8–18] including 2,703 patients with 426 end point events evaluated the association of crescent formation with kidney survival in IgAN. Nine studies [6, 11, 13, 17, 18, 20–23] showed comparison of kidney function between IgA with C0 and C1 (defined as absence and any presence of crescents, respectively, in the Oxford Classification; eGFR in 7 and urinary protein excretion in 3), 4 studies [6, 11, 20, 24] reported the two main treatments (immunosuppressive therapy in 4 and renin-angiotensin system blockades, abbreviated as RASBs, in 3), and 4 studies [17, 25–27] reported the association with other four pathologic lesions (M, E, S and T). Characteristics of the included studies are summarized in Table 1.

**Table 1:** Characteristics of included studies in this systematic review

Validation cohort study								
	Oxford cohort[6]	Joh[8]	Bazzi[22]	Walsh[9]	Katafuchi[10]	Shi[11]	Shima[12]	Edstrom Halling[13]
Country	Multicountry	Japan	Italy	Canada	Japan	China	Japan	Sweden
Ethnicity								
White	66%	—	NA	NA	—	—	—	NA
Asian	27%	100%	NA	NA	100%	100%	100%	NA
African	3%	—	NA	NA	—	—	—	NA
other	4%	—	NA	NA	—	—	—	NA
Design^a^	Multi	Single	Single	Multi	Single	Single	Multi	Single
No. pts	265	233	168	146	702	410	161	99
F/U (mo)	69	127	60±40	69.6	62	38	54	156
Age (y)	30 (4-73)	36 (18-70)	NA	NA	30 (8-82)	31	11.7	9.7
Age < 18 y	22.30%	0%	NA	0%	NA	0%	100%	100%
M:F	2.6:1	01:01	NA	1.74:1	01:01.2	01:01	1.7:1	1.4:1
Proteinuria (g/d)	1.7	1.3	NA	NA	0.85^b^	1.7	0.7	2
eGFR (mL/min/1.73m2)	83	78	NA	NA	82	85.8	103	100
CKD1/2/3/4/5^c^	36/38/26/0/0	27/50/23/0/0	NA	NA	37/37/21/3/1	43/38/19/0/0	NA	75/16/4/3/2
MAP (mm Hg)	98	94	NA	NA	92	94	79	85.4
HTN	31%	9%	54%	NA	NA	NA	NA	NA
RAAS blockade	74%	77%	NA	46%	37%	86.10%	NA	86%
Immunosuppressive therapy	29%	35%	NA	5.10%	32%^d^	42.70%	16%	43%
End point definition	50% decline in eGFR or ESKD; slope of eGFR	50% decline in eGFR or ESKD; slope of eGFR	Doubling of SCr or ESRD	Doubling of SCr, ESRD or death	ESKD	ESKD	CKD stages 3-5	50% decline in eGFR or ESKD
No. end point events	NA	58	NA	40	84	30	7	18
No. of pathologists	5, blinded	NA	NA	1, blinded	NA	2, blinded	NA	1, blinded
Lesion								
M0/M1	20/80	NA	NA	NA	88/12	44/56	36/64	69/31
E0/E1	58/42	NA	NA	NA	58/42	43/57	42/58	90/10
S0/S1	NA	NA	NA	NA	21/79	25/75	92/8	77/23
T0/T1/T2	NA	NA	NA	NA	71/18/12	78/14/8	99/1/0	84/12/3
C0/C1	55/45	NA	75/25	75/25	37/63	40/60	48/52	82/18
Crescent %^e^	9^f^	NA	19	5-60^f^	4.2 (0-73.3) f	16^f^	9.2	4.5
Adjusted factors	Initial GFR, MAP, proteinuria; initial GFR, F/U MAP, proteinuria	Initial GFR, MAP, proteinuria; initial GFR, F/U MAP, proteinuria	None	Age, gender, initial SBP, proteinuria, creatinine	Initial GFR, MAP, proteinuria, therapy (steroid or not), M,E,S,T	Initial GFR, MAP, proteinuria	Initial proteinuria	Initial proteinuria, 1 y-F/U proteinuria

Validation Cohort Study											
Kataoka[14]	Le[20]	Zeng[21]	Lv[15]	Zhang[25]	Rafalska[16]	Lee[17]	Kaneko[18]	Chan[24]	Li[23]	Stangou[26]	Stefan[27]
Japan	China	China	China	China	Poland	Korea	Japan	China	China	Greece	Romania
—	—	—	—	—	NA	—	—	—	—	NA	NA
100%	100%	100%	100%	100%	NA	100%	100%	100%	100%	NA	NA
—	—	—	—	—	NA	—	—	—	—	NA	NA
—	—	—	—	—	NA	—	—	—	—	NA	NA
Single	Multi	Multi	Multi	NA	Single	Single	Single	NA	Single	Single	NA
43	218	1,026	113	539	52	430	314	115	24	50	121
120	56	53	23.7	36	>6	61	106	NA	NA	68	NA
40	14 (2-17.9)	34	36.3	NA	32.5	34.9	36	NA	NA	40.7	40.1
0%	100%	0%	NA	NA	0%	0%	NA	NA	NA	NA	0%
1.5:1	1.9:1	01:01	1.4:1	NA	1.4:1	1.1:1	0.92:1	0.69:1	NA	2.6:1	2.2:1
1.8	1.5	1.3	4.44	NA	1.35	0.8^b^	1.05	NA	NA	1.5	NA
78.3	134	85	NA	NA	74.7	80.5	80.3	NA	NA	64.3	47
NA	NA	41/35/24/0/0	NA	NA	NA	36.7/42.8/19.3/1.2	NA	NA	NA	NA	NA
102.6	88	98	118.1	NA	97	NA	91.1	NA	NA	NA	NA
NA	NA	30.50%	69.90%	NA	NA	21.90%	NA	39.10%	NA	86%	NA
58.10%	61.50%	89%	69.00%	NA	NA	83.00%	59.55%	NA	NA	NA	98%
51.20%	56%	31%	47.80%	NA	NA	2.30%	60.19%	43.50%	NA	NA	49%
>150% increase of baseline SCr	50% decline in eGFR or ESKD	50% decline in eGFR or ESKD; slope of eGFR	ESRD	ESRD	>50% increase of SCr	50% decline in eGFR	50% decline in eGFR or ESKD	Doubling of SCr or starting of RRT	NA	ESRD	Doublingof SCr or ESRD
16	24	159	63	56	8	59	43	NA	NA	8	34
2, blinded	2	2	NA	NA	NA	1, blinded	5	NA	NA	NA	1
19/81	55/45	57/43	NA	NA	NA	41.6/58.4	57/43	NA	NA	38/62	NA
47/53	77/23	89/11	NA	NA	NA	86.3/13.7	80/20	NA	NA	74/26	NA
19/81	38/62	17/83	NA	NA	NA	42.3/57.7	33/67	NA	NA	76/24	NA
NA	93/6/1	72.7/24/3.3	NA	NA	95.2/4.8	63.3/26.5/10.2	93/5/2	NA	NA	64/20/16	NA
47/53	56/44	52/48	NA	58/42	83.3/16.7	81.2/18.8	59/41	78/22	58/42	82/18	69/31
NA	9^f^	8^f^	66.4	NA	NA	NA	NA	NA	NA	5-30^f^	NA
Age, sex, eGFR	Initial GFR, MAP, proteinuria	Initial GFR, MAP, proteinuria; initial GFR, F/U MAP, proteinuria	None	NA	Initial eGFR, MAP, proteinuria, tonsillectomy, S,T	Sex, age, initial eGFR, MAP, proteinuria, M,E,S,T	Iinitial eGFR, MAP, proteinuria, M,E,S,T, arteriolar hyalinosis	Sex, age, initial creatinine, proteinuria, HTN, DM, therapy (RASBs, steroid)	None	None	NA

Note: Values for continuous variables are given as mean or mean (range).

Abbreviations: CKD: chronic kidney disease; DM: diabetes mellitus; E: endocapillary hypercellularity; eGFR: estimated glomerular filtration rate; ESKD: end-stage kidney disease; F/U: follow-up; HTN: hypertension; M: mesangial hypercellularity; MAP: mean arterial pressure; NA: not available; pts: patients; RAAS: renin-angiotensin-aldosteronesystem; RRT: renal replacement therapy; S: segmental glomerulosclerosis; SCr: serum creatinine; T: tubular atrophy/interstitial fibrosis.

^a^Number of centers.

^b^Grams of protein per gram of creatinine.

^c^Percentage number indicates CKD stage.

^d^Steroid.

^e^The percentage of glomeruli with crescents.

^f^These values are given as median, range or median (range).

### Primary outcome

#### Kidney survival

**Figure 2:**
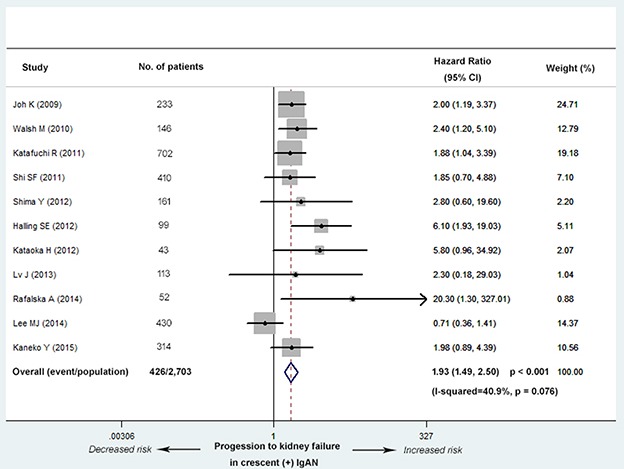
Hazard ratios (HR) for kidney failure for patients with versus without cellular/fibrocellular crescents The IgAN patients with crescents had an increased risk of worse kidney outcome (C0 as reference; HR, 1.93; 95% CI, 1.49-2.50; *P* < 0.001), with no evidence of heterogeneity (*I^2^* = 40.9%; *P* = 0.076).

Eleven studies (8 Asian, 2 European and 1 North American) with 2,703 patients and 426 end point events reported the association of crescent formation with kidney survival. These studies showed that a score of C1 was associated strongly with progression to kidney failure in a fixed-effects model (C0 as reference; hazard ratio, abbreviated as HR, 1.93; 95% confidence interval, abbreviated as 95% CI, 1.49-2.50; *P* < 0.001; Figure 2), with no evidence of heterogeneity (*I^2^* = 40.9%; *P* = 0.076).

The analyses for Asian and European populations were also conducted separately. The result indicated that ethnicity had no obvious impact on our conclusion (C0 as reference; Asia, HR, 1.69; 95% CI, 1.27-2.25; *P* < 0.001; Europe and North America, HR, 3.42; 95% CI, 1.88-6.21; *P* < 0.001; Supplementary Figure 1), with no evidence of heterogeneity separately (Asia, *I^2^* = 23.1%; *P* = 0.246; Europe and North America, *I^2^* = 42.8%; *P* = 0.174). Meanwhile, based on 3 confounders from Supplementary Table 1 (age, the percentage of patients under the age of 18 and use of immunosuppressive therapy), eight studies with more consistent baseline characteristics were included in a new meta-analysis. This analysis also verified the conclusion above (C0 as reference; HR, 2.13; 95% CI, 1.59-2.85; *P* < 0.001; Supplementary Figure 2), with no evidence of heterogeneity (*I^2^* = 0.0%; *P* = 0.757).

### Secondary outcome

#### eGFR and proteinuria levels

**Figure 3:**
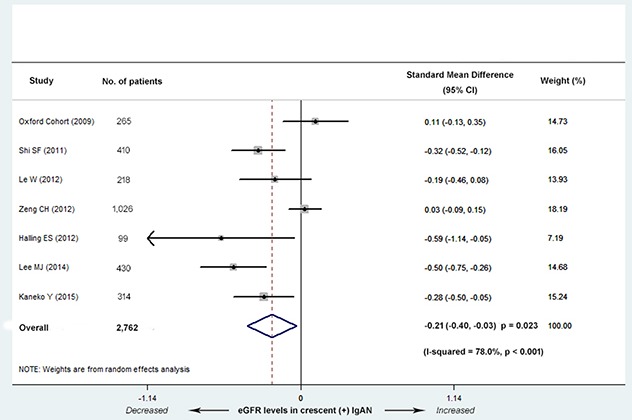
Standard mean differences (SMD) for the level of eGFR for patients with versus without cellular/fibrocellular crescents The IgAN patients with crescents had decreased eGFR levels (SMD, -0.21; 95% CI, -0.40--0.03; *P* = 0.023), with high heterogeneity (*I^2^ =* 78%; *P* < 0.001).

Seven studies (5 Asian and 2 European) with 2,762 patients reported a difference in eGFR levels between IgAN with and without crescents. These studies showed that the eGFR level was significantly decreased in C1 group (standard mean difference, abbreviated as SMD, -0.21; 95% CI, -0.40--0.03; *P* = 0.023; Figure 3). Evidence of high heterogeneity lay in the magnitude of the effect across the included studies (*I^2^ =* 78.0%; *P* < 0.001). Univariate meta-regression analysis revealed the presence of heterogeneity mainly in the effect of design type (multi-center or single-center cohort, coefficient= 0.41; 95% CI, 1.02-2.24; *P* = 0.042) and ethnicity (Asian or not, coefficient= -0.65; 95% CI, 0.29-0.93; *P* = 0.033) (Table 2).

**Table 2:** Univariate metaregression analysis of possible sources of heterogeneity across studies

Possible source of heterogeneity	Adjusted-R2 (%)a	Pb
No. of patients	0.3	0.301
Ethnicity (Asian or not)	61.78	0.033
Study design (multi- or single-center cohort)	87.86	0.042
Age	−9.30	0.310
Age < 18 y (%)	−28.77	0.627
Follow-up time (months)	14.74	0.152
Male/female	−10.84	0.465
Hypertension (%)	−14.07	0.281
Immunosuppressive therapy (%)	−35.70	0.926
Treatment with RASBs (%)	−36.04	0.682
End point event^c^ number	−2.84	0.351

^a^Proportion of between-study variance explained by covariates.

^b^P value derived from the joint test for all covariates with Knapp–Hartung modification.

^c^End-stage kidney disease (ESRD), >50% decrease in estimated glomerular filtration rate (eGFR), or doubling of serum creatinine concentration.

**Figure 4:**
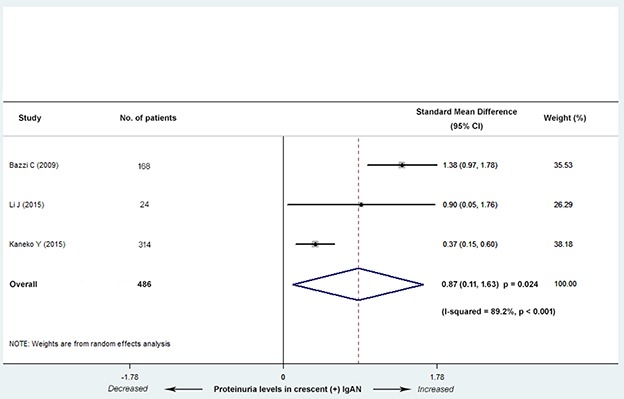
Standard mean differences (SMD) for the level of proteinuria for patients with versus without cellular/fibrocellular crescents The IgAN patients with crescents had increased proteinuria levels (SMD, 0.87; 95% CI, 0.11-1.63; *P* = 0.024), with high heterogeneity (*I^2^ =* 89.2%; *P* < 0.001).

Three studies (2 Asian and 1 European) with 486 patients reported a difference in the proteinuria level between C0 and C1 group. These studies showed that the proteinuria level was significantly increased in C1 group (SMD, 0.87; 95% CI, 0.11-1.63; *P* = 0.024; Figure 4). Evidence of high heterogeneity was noted in the magnitude of the effect across the included three studies (*I^2^* = 89.2%; *P* < 0.001). The number of included studies was too small to perform the univariate meta-regression analysis.

### Use of immunosuppressive therapy and RASBs

**Figure 5:**
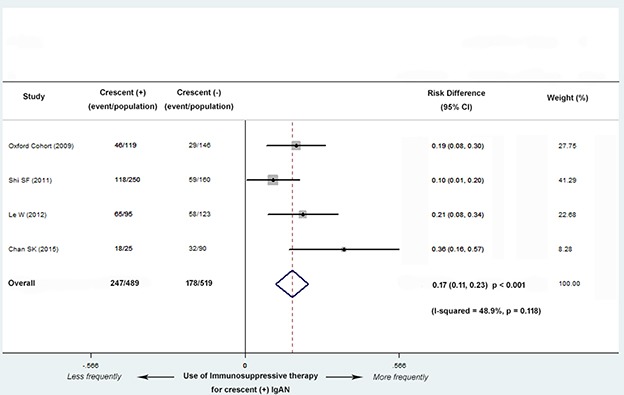
Risk differences (RD) for immunosuppressive therapy for patients with versus without cellular/fibrocellular crescents The IgAN patients with crescents were more likely received immunosuppressive treatment (RD, 0.17; 95% CI, 0.11-0.23; *P* < 0.001), with no evidence of heterogeneity (*I^2^* = 48.9%; *P* = 0.118).

Four studies (3 Asian and 1 European) with 1,008 patients reported the different use of immunosuppressive therapy between C0 and C1 group. These studies showed that patients with C1 were more likely received immunosuppressive treatment in a fixed-effects model (risk difference, abbreviated as RD, 0.17; 95% CI, 0.11-0.23; *P* < 0.001; Figure 5), with no evidence of heterogeneity (*I^2^* = 48.9%; *P* = 0.118).

**Figure 6:**
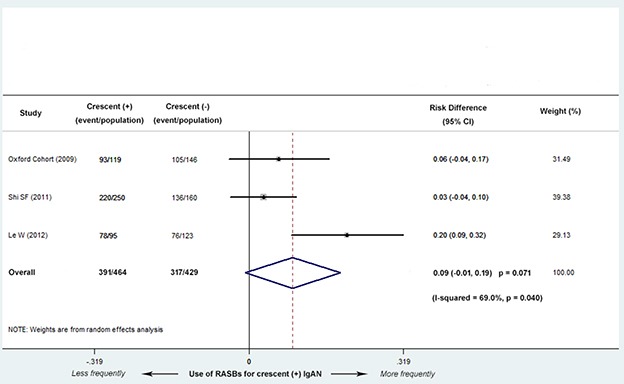
Risk differences (RD) for RASBs treatment for patients with versus without cellular/fibrocellular crescents There were no significant differences in the use of RASBs between the two groups (RD, 0.09; 95% CI, -0.01-0.19; *P* = 0.071), with evidence of heterogeneity (*I^2^* = 69.0%; *P* = 0.040).

Three studies (2 Asian and 1 European) with 893 patients reported the different use of RASBs treatment between C0 and C1 group. There were no significant differences in the use of RASBs between the two groups (RD, 0.09; 95% CI, -0.01-0.19; *P* = 0.071; Figure 6), with moderate evidence of heterogeneity (*I^2^* = 69.0%; *P* = 0.040).

### Association with other lesions

**Figure 7:**
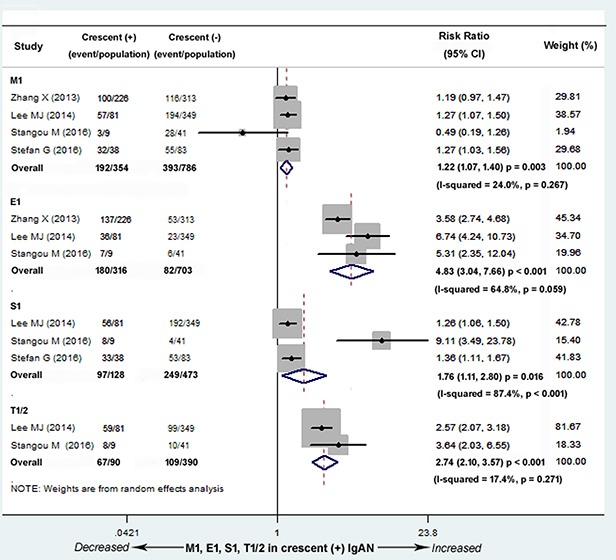
Risk ratios (RR) for other lesions for patients with versus without cellular/fibrocellular crescents The numbers of patients with M1 (RR, 1.22; 95% CI, 1.07-1.40; *P* = 0.003), E1 (RR, 4.83; 95% CI, 3.04-7.66; *P* < 0.001), S1 (RR, 1.76; 95% CI, 1.11-2.80; *P* = 0.016), and T1/2 (RR, 2.74; 95% CI, 2.10-3.57; *P* < 0.001) were significantly larger in the IgAN patients with crescents, with no evidence of heterogeneity in M, E, T (M1, *I^2^* = 24.0%; *P =* 0.267; E1, *I^2^* = 64.8%; *P =* 0.059; *I^2^* = 17.4%; *P* = 0.271), and high heterogeneity in S1 (*I^2^* = 87.4%; *P* < 0.001). M1 defined as M score > 0.5, E1 defined as any E lesion present, S1 defined as any S lesion present, T1/2 defined as > 25% but < 50% and > 50% T lesion present.

Four studies (3 Asian and 1 European) with 1,140 patients reported the different proportions of the four Oxford Classification pathologic lesions (M, E, S and T) between C0 and C1 group. These studies demonstrated that the number of patients with M1 (defined as M score > 0.5; risk ratio, abbreviated as RR, 1.22; 95% CI, 1.07-1.40; *P* = 0.003; Figure 7), E1 (defined as any E lesion present; RR, 4.83; 95% CI, 3.04-7.66; *P* < 0.001; Figure 7), S1 (defined as any S lesion present; RR, 1.76; 95% CI, 1.11-2.80; *P* = 0.016; Figure 7), and T1/2 (defined as > 25% but < 50% and > 50% T lesion present; RR, 2.74; 95% CI, 2.10-3.57; *P* < 0.001; Figure 7) were significantly larger in C1 group. There was no evidence of heterogeneity in the magnitude of the effect in M, E, T (M1, *I^2^* = 24.0%; *P =* 0.267; E1, *I^2^* = 64.8%; *P =* 0.059;*I^2^* = 17.4%; *P*= 0.271), and high heterogeneity in S1 (*I^2^* = 87.4%; *P* < 0.001). The number of included studies in S was not sufficient to perform the univariate meta-regression analysis.

### Publication bias

**Figure 8:**
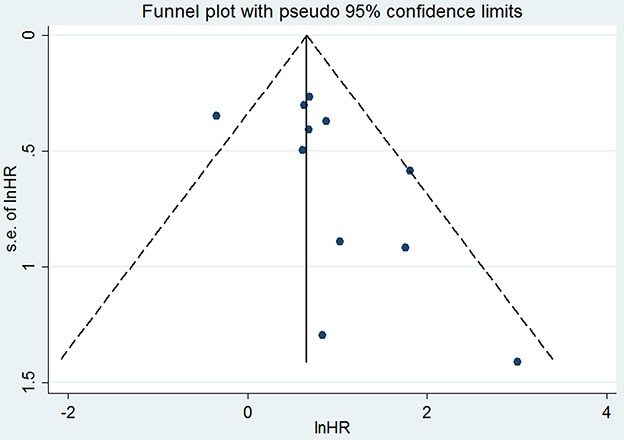
Funnel plot for testing the publication bias of the 11 studies evaluating the association between crescent formation and kidney survival No obvious publication bias affected the association of crescent formation in IgAN with kidney survival. HR: hazard ratio; s.e.: standard error.

No obvious asymmetry was observed in the funnel plot (Figure 8). Furthermore, the results of Begg's test (*P* = 0.119) and Egger's test (*P* = 0.100) indicated no obvious publication bias among the 11 studies accessing the association of crescent formation in IgAN with kidney survival.

## DISCUSSION

In the 2009 Oxford Classification, four pathologic lesions were proposed as independent prognostic markers in IgAN: mesangial hypercellularity (M), endocapillary hypercellularity (E), segmental glomerulosclerosis (S), and tubular atrophy and interstitial fibrosis (T). This classification has been validated in numerous studies with different ethnic populations [7, 10–14, 16, 18, 28]. However, the predictive value of crescent lesions for worse kidney survival was not determined mostly due to the low prevalence of crescents in included patients and the lack of end point events [6].

Systematic review and meta-analysis is an appropriate strategy to overcome the limitations noted above. In the present study with 2,703 patients and 426 end point events, we found that crescents were associated strongly with the progression to kidney failure. Compared with C0 group, C1 group had an increased risk of worse kidney outcome, with no evidence of heterogeneity across all included studies. A large number of crescent lesions (>50% of all glomeruli) often lead to rapidly progressive renal failure in IgAN patients [6, 29, 30]. In addition, the prognostic value of moderate crescent formation (<50% of all glomeruli) in IgAN remains controversial [31, 32]. In this meta-analysis, we were unable to perform a subgroup analysis to explore the predictive significance of mild, moderate or severe crescent formation in IgAN respectively. It was because only 5 of the 20 included studies [10, 12, 16–18] had the exact definitions of C1 according to the percentage of glomeruli with crescent lesions, and the definitions were inconsistent among these studies. The JHC 2013 of IgAN confirmed crescent formation as the predictive marker of ESKD and developed four histological grades (HGs) established corresponding to the percentage of glomeruli exhibiting cellular or fibrocellular crescents, global sclerosis, segmental sclerosis or fibrous crescents, respectively [19], but we were unable to determine the precise proportion of crescent lesions in each grade. Therefore, the accurate degree of crescent formation with prognostic value in IgAN should still be further explored.

It is notable that our study also explored some secondary clinical outcomes not included in other similar meta-analyses [28]. First, the patients with crescents had a significantly increased proteinuria level and reduced eGFR level than those without this lesion. Using meta-regression we identified the interaction among study design (multi-center or single-center cohort), ethnicity (Asian or not) and eGFR level. This finding might be attributed to variable baseline levels of renal biomarkers in different ethnic populations. Second, the patients with crescents received immunosuppressive therapy more frequently. However, whether crescent lesions were more responsive to immunosuppressant or RASBs treatment is not known due to the limited number of studies [11, 18, 33]. Finally, the numbers of patients with M1, E1, S1 and T1/2 lesions were considerably increased in C1 group. These results suggested crescent formation as a predictive factor of worse renal outcome from another clinical aspect.

This meta-analysis has some strengths. On one hand, a wide range of IgAN populations with crescents were included. The number of patients was approximately 20-fold increased compared with that of the 2009 Oxford cohort and supported the use of crescent lesions in the classification of IgAN. On the other hand, some renal biomarkers, M, E, S, T and treatment strategies were observed as secondary outcomes of crescent formation in IgAN, which had not been described in previous meta-analyses. There are also some limitations in the present study. First, all the included studies were retrospective. This study was not an individual patient-data meta-analysis, so we could not evaluate the interaction between crescent lesions and their response to two main treatments. Second, the number of included studies for evaluating secondary renal outcomes was too small. This limitation could explain the high heterogeneity among the included studies for evaluating the level of eGFR, proteinuria and the number of patients with S1 lesions. Finally, the quality of the included studies was variable. Unlike randomized clinical trials, a reliable and robust tool is not currently available to evaluate the risk of bias in nonrandomized studies. Thus, we could not evaluate the effect of study quality on our pooled data. To reduce the possibility of publication bias, we also searched conference proceedings and consulted experts to identify unpublished studies.

In conclusion, our study confirms that crescent lesions in IgAN are associated strongly with progression to kidney failure. In addition, IgAN patients with crescents exhibit increased proteinuria levels, reduced eGFR levels, and a larger number of patients with M1, E1, S1 and T1/2 lesions and received immunosuppressive therapy more frequently. Therefore, our finding supports the addition of crescent lesions to the new Oxford Classification to identify IgAN patients with an increased rate of progression to ESKD.

## MATERIALS AND METHODS

### Data sources, search strategy, and selection criteria

The PRISMA (Preferred Reporting Items for Systematic Reviews and Meta-analyses) was used as a guide in our study to ensure a standard approach for transparent and complete reporting of systematic reviews and meta-analyses [34]. Relevant studies were identified by searching PubMed and EMBASE (from January 2009 to July 2016) with medical subject heading terms and text words as follows in multiple combinations: “IGA Nephropathy,” “IGA Type Nephritis,” “Nephropathy, Immunoglobulin A,” “Immunoglobulin A Nephropathy,” “IGA Glomerulonephritis,” “Glomerulonephritides, IGA,” or “Berger Disease”; “Extracapillary proliferation,” “Extracapillary hypercellularity,” or “Crescent”. No language or region restrictions were imposed. In addition, other relevant studies were also identified through manual searches of the reference lists and bibliographies.

All published articles that met the following inclusion criteria were included in this meta-analysis: primary IgAN classified as C0 and C1; study end points included ESKD, >50% decrease in eGFR, or doubling of serum creatinine concentration; reports showed available data of kidney outcomes including the level of eGFR (ml/min/1.73m^2^), urinary protein excretion (g/d) and the number of patients with other lesions (defined as M, E, S and T in the Oxford Classification); reports disclosed the number of patients using the immunosuppressive therapy or RASBs. Studies meeting the following criteria were excluded: duplication, basic research studies, case reports, nonoriginal studies including reviews, commentaries, letters and editorials, and studies that did not investigate crescent formation as a variable or clinical characters outcomes. The literature search, data extraction, and validity assessment were performed by 2 coauthors (B.L. and L.C.).

### Data extraction and quality assessment

Published reports were obtained for eligible studies, and standardized information was presented in Table 1. The extracted data were as follows: participants’ characteristics, follow-up duration, mean arterial blood pressure (MAP), renal function measurements (eGFR and 24-hour urine protein excretion), number and percentage of patients receiving treatment (immunosuppressive therapy and RASBs), number of end point events, pathologic methodology, characteristics of the Oxford Classification pathologic lesions (M, E, S and T) and crescent lesions, and statistical methodology. Disagreements about the extracted data were adjudicated by a third reviewer (X.S.) to make a final decision.

### Statistical analysis

All of the statistical analyses were performed using Stata, version 12.0 (StataCorp LP). Individual-study HRs, RRs and RDs with 95% CIs were calculated or extracted from each study for dichotomous outcomes and SMD for continuous outcomes. The heterogeneity among the studies was examined with the *I^2^* statistics. The potential heterogeneity in the predictive value of crescent formation was explored by comparing the summary results obtained from the studies: number of patients, ethnicity, study design, age, follow-up time, gender, hypertension, immunosuppressive and RASBs treatment, and number of end point events. Fixed-effects models (Inverse Variance or Mantel-Haenszel) were used for low heterogeneity, otherwise, the random effect models were used. Moreover, publication bias was assessed by Egger's test and Begg's test among the 11 studies evaluating the association of crescent formation with kidney survival. A two-tailed *P* < 0.05 was considered statistically significant for all analyses.

## SUPPLEMENTARY MATERIALS FIGURES AND TABLES


