# Supplementary material for: Evaluation of crescent formation as a predictive marker in immunoglobulin A nephropathy: a systematic review and meta-analysis
Source: Oncotarget. 2017 Apr 28;8(28):46436–48. doi: 10.18632/oncotarget.17502 (PMC5542279; doi:10.18632/oncotarget.17502)
Supplement: Supplementary file 1 [file oncotarget-08-46436-s001.pdf]

Evaluation of crescent formation as a predictive marker in immunoglobulin A nephropathy: a systematic review and meta-analysis

SUPPLEMENTARY MATERIALS

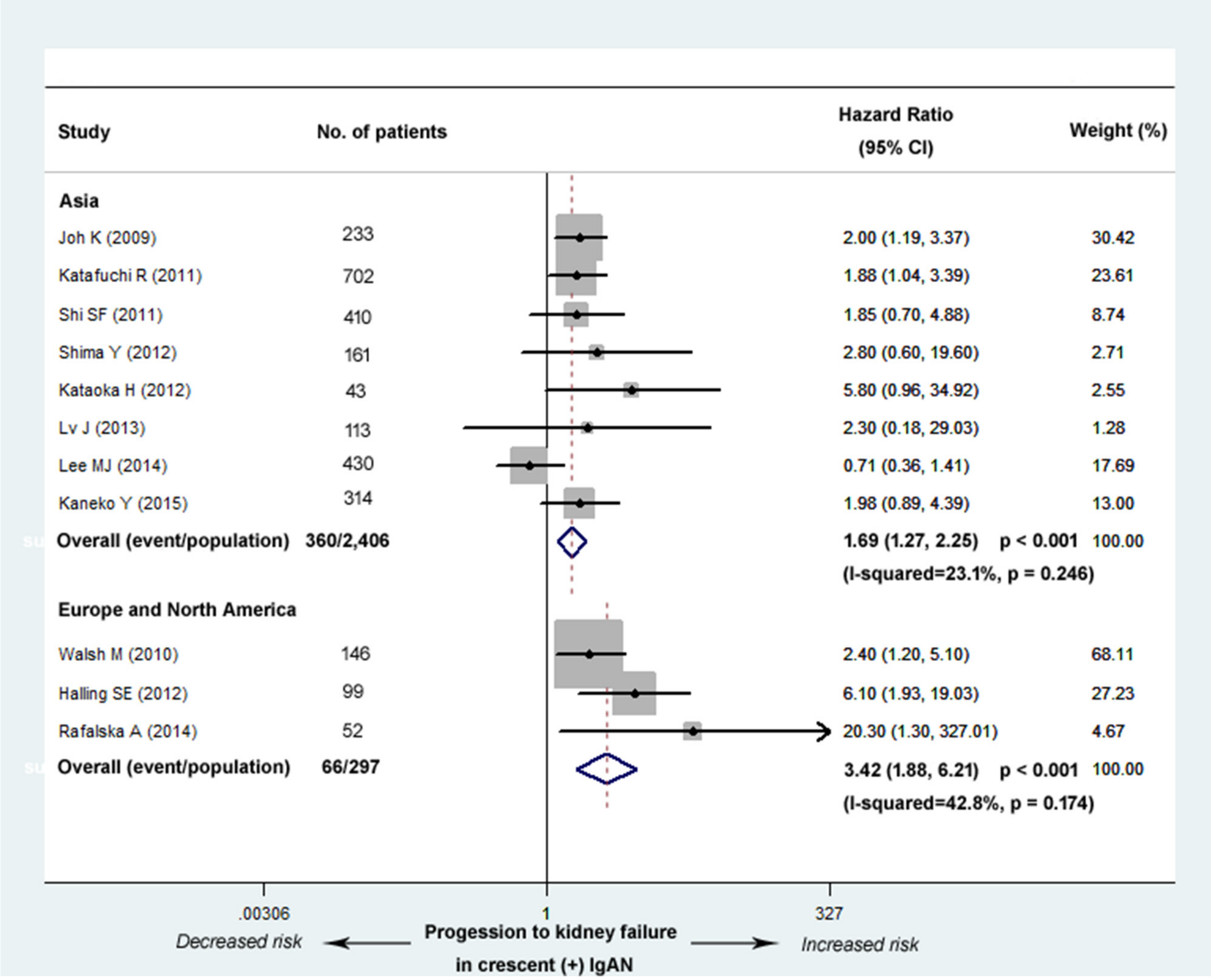

Supplementary Figure 1: Hazard ratios (HR) for kidney failure for patients with versus without cellular/fibrocellular crescents in Asian studies and European and North American studies separately. The IgAN patients with crescents in Asian studies and European and North American studies had an increased risk of worse kidney outcome separately (C0 as reference; Asia, HR, 1.69; 95% CI, 1.27-2.25; $P < 0.001$; Europe and North America, HR, 3.42; 95% CI, 1.88-6.21; $P < 0.001$), with no evidence of heterogeneity separately (Asia, $I^2 = 23.1\%$; $P = 0.246$; Europe and North America, $I^2 = 42.8\%$; $P = 0.174$).

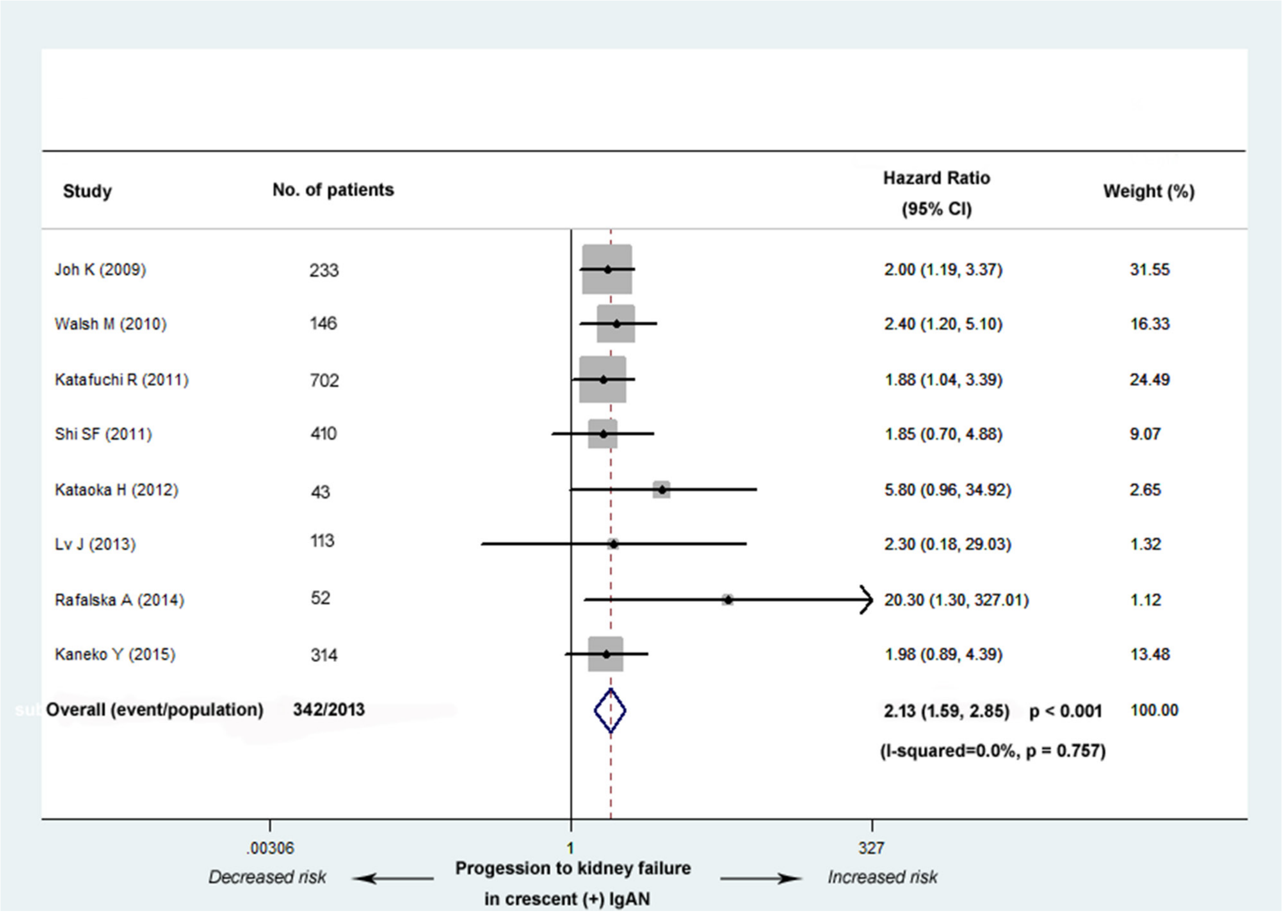

Supplementary Figure 2: Hazard ratios (HR) for kidney failure for patients with versus without cellular/fibrocellular crescents in the 8 included studies. The IgAN patients with crescents in the 8 studies with more consistent baseline characteristics had an increased risk of worse kidney outcome (C0 as reference; HR, 2.13; 95% CI, 1.59-2.85; $P < 0.001$), with no evidence of heterogeneity separately ($I^2 = 0.0\%$; $P = 0.757$).

Supplementary Table 1: Univariate metaregression analysis of possible confounders across studies

Possible source of heterogeneity	Adjusted-R^2 (%)[a]	*P*[b]
No. of patients	0.67	0.114
Ethnicity (Asian or not)	30.56	0.104
Study design (multi- or single-center cohort)	-21.06	0.727
Age	43.80	0.190
Age < 18 y (%)	34.73	0.267
Follow-up time (months)	22.25	0.151
Male/Female	-8.73	0.230
eGFR (mL/min/1.73m^2)	29.84	0.343
Immunosuppressive therapy (%)	34.06	0.166
Treatment with RASBs (%)	-36.02	0.735
End point event[c] number	14.85	0.091
Lesion (%)		
M1	-35.17	0.902
E1	-25.76	0.697
S1	-7.8	0.492
T1+T2	2.27	0.214
C1	-39.96	0.916

C: Crescents; E: endocapillary hypercellularity; eGFR: estimated glomerular filtration rate; M: mesangial hypercellularity; T: tubular atrophy/interstitial fibrosis; RASBs: renin-angiotensin system blockades.

[a]Proportion of between-study variance explained by covariates.

[b]P value derived from the joint test for all covariates with Knapp–Hartung modification.

[c]End-stage kidney disease (ESRD), >50% decrease in estimated glomerular filtration rate (eGFR), or doubling of serum creatinine concentration.
